# Peer counselling improves breastfeeding practices: A cluster randomized controlled trial in urban Bangladesh

**DOI:** 10.1111/mcn.12605

**Published:** 2018-04-16

**Authors:** Gulshan Ara, Mansura Khanam, Nowshin Papri, Baitun Nahar, Md Ahshanul Haque, Iqbal Kabir, Michael J. Dibley

**Affiliations:** ^1^ ICDDR, B Dhaka Bangladesh; ^2^ Bangladesh Breastfeeding Foundation (BBF) Dhaka Bangladesh; ^3^ Sydney School of Public Health, Edward Ford Building (A27) University of Sydney Sydney New South Wales Australia

**Keywords:** early initiation of breastfeeding, exclusive breastfeeding, intervention, peer counselling, randomized controlled trial

## Abstract

This study aimed to evaluate the impact of peer counselling on early initiation of breastfeeding (EIBF) and exclusive breastfeeding (EBF) rates for mother–infant pairs living in urban slums, Dhaka, Bangladesh. This randomized controlled trial enrolled 350 mother–infant pairs from selected slums between September 2014 and July 2016. The women assigned to intervention group received peer counselling from locally recruited, trained community female volunteers starting in third trimester of pregnancy until 6 months after delivery; control group received no intervention. EIBF, any liquids given after birth, and EBF were compared between groups. Follow‐up was scheduled at enrolment, following childbirth, and every 2 months up to 6 months after delivery. Multiple logistic regressions were used to assess the effect peer counselling and other associated factors on EIBF and EBF practices. EIBF rate was higher in the intervention group than in the control group (89.1% vs. 77.4%, *p* = .005). More mothers in intervention group were exclusively breastfeeding at 5 months than mothers in the control group (73% vs. 27%, *p* < .005). Control mothers were twice as likely to not practice EIBF compared with intervention mothers (adjusted odds risk [aOR]: 2.53, CI [1.29, 4.97], *p* = .007). Overall, caesarean section was associated with an 8.9‐fold higher risk of not achieving EIBF (aOR: 8.90, CI [4.05, 19.55], *p* < .001). Intervention mothers were 5.10‐fold more likely to practice EBF compared with control mothers (aOR: 5.10, CI [2.89, 9.01], *p* < .001) at 5 months. This study demonstrates peer counselling can positively influence both EIBF and EBF among mothers living in urban area.

**Key messages**

- Peer counselling has a positive effect on initiation of breastfeeding within 1 hr of birth and exclusive breastfeeding practices.
- Emphasis on encouraging EIBF and discouraging prelacteal feeding should be focused on mothers living who delivered by caesarean section.

## 1 INTRODUCTION

Worldwide, approximately four million neonatal deaths occur annually within the first 4 weeks of life. The majority of these neonatal deaths are among infants in the developing world, mainly from preventable causes, and approximately half occur in the home. The benefits of breastfeeding for reducing neonatal and infant mortality are well documented (Victora & Barros, 2000). Therefore, promotion of early and exclusive breastfeeding (EBF) is a key intervention. Early initiation of breastfeeding (EIBF) is defined as putting the infant to the breast within 1 hr of birth. EBF is recommended by the World Health Organization (WHO), which states that infants should be breastfed exclusively from birth until 6 months of age, followed by continued breastfeeding alongside gradual introduction of solid foods (World Health Organization and UNICEF, 2003). Several beneficial effects of breastfeeding have been reported, including reductions in the incidence and severity of infectious diseases such as diarrhoea, respiratory tract infections, otitis media, and urinary tract infections, as well as reductions in the incidence of Types 1 and 2 diabetes mellitus, being overweight, obesity, and asthma (Gartner et al., 2005). Conversely, early introduction of breast milk substitutes or semi‐solid foods and delayed introduction of appropriate semi‐solid complementary foods are risk factors associated with the rapid increase in the prevalence of undernutrition among children between 6 and 24 months old (Ramachandran, 2004).

The beneficial effects of EIBF and EBF in the first month of life on mortality have recently been established. A large cohort study conducted in rural Ghana showed that EIBF (within 1 hr) was associated with a 22% reduction in neonatal deaths (Population Attributable Fraction [PAF] adjusted odds ratio [aOR], 41.3%; Edmond et al., 2006). Additionally, the risk of neonatal mortality was higher among infants without EBF practices compared with those with EBF. Improved breastfeeding during the neonatal period helps to reduce mortality and benefits the health, growth, and development of the child in the first year and beyond (Huffman, Zehner, & Victora, 2001). Establishment of good breastfeeding practices in the first days after birth is critical for successful breastfeeding practice and infant health. Initiation of breastfeeding is easiest and most successful when a mother is physically and psychologically prepared for birth and is informed, supported, and confident in her ability to breastfeed and care for the newborn (Development., A.f.E., 2003).

The importance of community support for EIBF was noted in a recent neonatal survival review published in *The Lancet* covering a range of potential interventions (including EIBF; Darmstadt et al., 2005). Community support via outreach and health education is important for the prevention of neonatal deaths in settings with high mortality and weak health systems to improve home‐care practices and increase the demand for skilled care and care seeking (Darmstadt et al., 2005). Community‐based interventions such as one‐to‐one or group counselling to promote and support infant and young child feeding, along with policy measures and improvements in facility‐based services, are strongly emphasized in the global strategy developed jointly by the WHO and United Nations Children's Fund (UNICEF). A recent meta‐analysis revealed delivery of individual peer counselling to mothers significantly increased the rates of EBF in both the neonatal period (15 studies; odds ratio [OR] = 3.45, 95% CI [2.20, 5.42], *p* < .0001) and at 6 months of age (nine studies; 1.93, [1.18, 3.15], *p* < .0001; Edmond et al., 2006). Studies conducted in various developing countries have shown the extent of malnutrition is more severe among children living in slums compared with children living in developed city areas and, sometimes, even compared with children living in rural regions (Fakir & Khan, 2015; Glewwe, Koch, & Nguyen, 2002). An increased risk of inappropriate child feeding practices has been reported among the rapidly growing slum populations in urban areas (Kumar, Nath, & Reddaiah, 1989), as many of these families lack the traditional support of the joint family system. Urban slums are a continually increasing phenomenon in South Asian countries and are underserved by medical facilities (Parvin, Ahsan, & Shaw, 2013). Therefore, we undertook a randomized controlled trial (RCT) to examine whether peer counselling can improve EIBF and EBF practices in an urban slum in Dhaka, Bangladesh.

## 2 RESEARCH DESIGN AND METHODS

### 2.1 Trial design

This prospective, RCT to examine the impact of peer counselling—starting in the third trimester of pregnancy and continuing until 6 months after delivery—on infant feeding practices was approved by the local ethics committee and registered at http://clinicaltrial.gov/ as NCT03040375 (last updated: January 31, 2017). Study participants were randomized into two groups: peer counselling (intervention) and no peer counselling (control).

### 2.2 Setting

The study area was Mirpur, a district of Dhaka with a total population of 5,580,000. The major source of income in Mirpur is wage labour (49.9%), and 53.0% of household heads have no education. The most common improved source of drinking water is a piped water connection inside the user's dwelling, plot or yard (62.8%), and 37.5% of the population of Mirpur have access to sanitary means of waste disposal. Antenatal check‐ups are most commonly performed by Non Government Organization (NGO) facilities (approximately 40%), followed by public sector health facilities (approximately 20%) and private clinics (approximately 10%). Local pharmacies are the most widely known health care facilities in Mirpur (Abul Barkat, Mahiyuddin, Poddar, Hossain, & Ahmed, 2015).

### 2.3 Study participants

Pregnant women aged between 16 and 49 years old with no more than three living children or a parity of five and who intended to reside in the area for at least 6 months after delivery were identified for the study from a house‐to‐house survey. Women with documented heart disease, insulin‐dependent diabetes mellitus, or pre‐eclampsia in a previous pregnancy were not included. Mother–infant pairs were excluded in cases with congenital anomalies, admission to intensive care, or a birthweight below 1.5 kg (Haider, Ashworth, Kabir, & Huttly, 2000).

### 2.4 Ethics approval and consent to participate

This study (PR‐14091) was approved by the Research Review Committee and Ethical Review Committee, the two compulsory components of the institutional review board of International Centre for Diarrhoeal Disease Research, Bangladesh (icddr,b). Written consent was provided by all study participants. The field research assistants informed the participants about the purpose of the study at the beginning of each interview by reading a consent form. Written consent was also taken from the parent/guardian for anthropometric measurement of the study infants. The respondents were informed of the important point that their participation was voluntary and they were allowed to withdraw themselves at any point of time during the interview/study. The respondents were also informed that the information obtained from this survey would be anonymous and have a broader impact, guide the development of policies and programs related to child nutrition, growth, and development, and would contribute to improve the child health services in Bangladesh and elsewhere.

### 2.5 Intervention

#### 2.5.1 Selection and training of peer counsellors

Women with personal breastfeeding experience, who had at least 8 years of schooling, who were motivated to help other mothers breastfeed, and who resided in the intervention area were selected to become peer counsellors. Peer counsellors were recruited by the International Centre for Diarrhoeal Disease Research, Bangladesh (icddr,b) and paid a monthly salary. The WHO/UNICEF Breastfeeding Counselling Course, which was validated in a previous study (Haider, Ashworth, Kabir, & Huttly, 2000), was adapted to the local language and culture and used to train peer counsellors. Training was provided over 40 hr (4 hr daily for 10 days). Counselling skills were taught by demonstration and role play, including listening to mothers, learning about their difficulties, assessing the position and attachment of the baby during breastfeeding, building mothers' confidence, giving support, and providing relevant information and practical help when required. One peer counsellor was recruited from each intervention cluster and was responsible for delivering the intervention to 30–35 mothers residing in the same area. Their performance was monitored at least three times over the study period by one breastfeeding supervisor based at the icddr,b field office.

#### 2.5.2 Counselling

The intervention group received at least 10 scheduled visits between the third trimester and 6 months after delivery: three in the last trimester of pregnancy, three in the first month after delivery (one within 48 hr of delivery, one 10–14 days after, and one 24–28 days after), and four visits in the second to sixth months. Counsellors were free to make and receive additional contact with the intervention group if the mothers required additional support. Counselling was given at home, and other family members were included. The duration of each visit was typically 20–40 min. During the two antenatal contacts, the peer counsellors emphasized to the mothers and other members of the family who would support her during delivery the importance of the mother holding the baby within a few minutes of delivery and gave instructions on how to initiate breastfeeding within 1 hr of delivery. They discouraged prelacteal feeds and the use of other fluids and foods after lactation was initiated. The peer counsellors encouraged the mothers to eat more of their usual foods to support lactation and to rest appropriately during the third trimester. These meetings also covered problems with breastfeeding that the mother may encounter and how best to address them.

Fathers and other household members were briefed on the importance of keeping the mother happy and joyful and not subjecting her to violence or harsh treatment. Fortnightly group meetings were organized with the participation of pregnant women, lactating mothers, and their family members. In these meetings, mothers and family members were encouraged to take extra care of the pregnant woman to ensure proper nutrition, attendance of antenatal check‐ups, proper intake of iron tablets, safe delivery, delivery at health facilities, colostrum feeding, and EIBF and continuation of EBF. The peer counsellors emphasized the negative effects of prelacteal feeding, breast milk substitutes, and early introduction of complementary feeding. The peer counsellors provided frequent home visits to support the mothers with EBF.12

### 2.6 Sample size

The number of mother–infant pairs required was calculated based on achieving a difference of least 20 percentage points in the prevalence of EBF between the intervention and control groups, with a 5% significance level, 80% power, and design effect of 1.5 in a two‐tailed test. The expected prevalence of EBF in the control group was 27% (from unpublished data from another RCT in Mirpur area, personal communication with M. J. Dibley et al.). A sample size of 156 mother–infant pairs was calculated; due to the nature of community‐level interventions, expected migration rate of 12%, and potential loss to follow‐up, the estimated sample size was increased to 175 mother–infant pairs for each group.

#### 2.6.1 Randomization

The trial was conducted in two wards of Mirpur municipality. The average population of a ward is 350,000. There are approximately five wards, each with an average population of 70,000. The interviewer visited door‐to‐door to identify pregnant women in their third trimester at every 2 months interval. Women who fulfilled the inclusion criteria and provided informed consent were randomly allocated to either the intervention or control group.

### 2.7 Collection of data

Four female field research assistants were recruited and trained over 2 weeks. Each mother was visited by an interviewer four times. Structured questionnaires with precoded closed questions were used during the home interviews. Data on socio‐economic and demographic variables, maternal and pregnancy factors, and previous infant feeding were obtained at enrolment. Details of delivery and early feeding were obtained within 72 hr of birth (60–80 hr). Data on feeding practices were collected every 2 months. To ensure the quality of the data, 10% of the interviews were observed by the field research supervisor. The data were separately recorded, entered, and analysed to ensure consistency with the data collected by the field research assistants. If any inconsistencies were observed, the principal investigator revisited and re‐interviewed the same subjects using the same questionnaire. Unscheduled field visits and spot checks by the principal investigator and research physician were also performed to monitor the quality of data collection.

#### 2.7.1 Data analysis

Data were entered into STATA v13 (StataCorp; College Station, TX, USA). The primary analyses compared the prevalence of EIBF and EBF in children at 0–6 months of age using Pearson's chi square test to calculate 95% confidence intervals for the group difference. Secondary outcome variables included the proportion of mothers who fed colostrum or prelacteals after delivery. For the primary trial outcome, the results of two‐sided 5% tests are reported.

Bivariate and multivariable logistic regressions were used to identify predictive variables, and ORs with 95% confidence intervals and value were used to measure the strength of the associations. Variables with significant associations (aOR, 95% confidence intervals [CI], *p* value < .05) were included in the final regression model; variables with a *p* value < .05 were defined as predictor variables.

## 3 RESULTS

### 3.1 Baseline comparison

**Figure 1:**
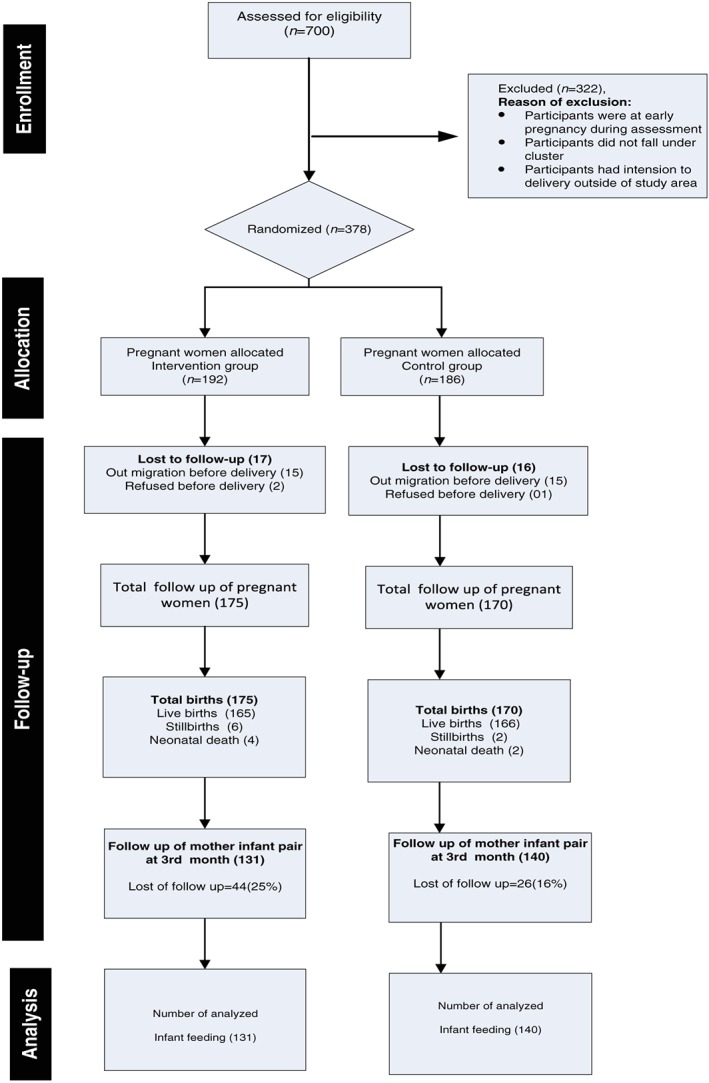
Trial profile

Of 700 pregnant women contacted, 378 were enrolled in the study. Of the 322 (46%) women excluded, the main reasons were that it was too early in the pregnancy, the mother did not live in the cluster, or the mother intended to deliver her child outside of the study area **(**Figure 1 **)**. At recruitment, participants did not know if they would be receiving the services of an infant feeding counsellor. The households in the intervention and control groups were similar: Over 80% of mothers in both groups used piped water as their source of drinking water (97.7% and 81.8% in intervention and control groups, respectively; *p* = .000). Over three fourths of mothers lived in one‐room houses (82.3% and 75.3% in intervention and control groups, respectively; *p* = 0.332). The majority of households in both groups used water‐sealed sanitary latrines. Most of the mothers in both groups were housewives (Table 1)**.**

**Table 1:** Sociodemographic characteristics of the mothers by study group

	Intervention (175)	Control (170)	*p* value
	*n* (%)	*n* (%)	
Main source of drinking water			
Tube well	4 (2.3)	31 (18.2)	
Supply (piped)	171 (97.7)	139 (81.8)	.000
Number of sleeping rooms in the household			
One	144 (82.3)	128 (75.3)	
Two	20 (11.4)	29 (17.1)	.332
Three	7 (4.0)	6 (3.5)	
Four or more	4 (2.3)	7 (4.1)	
Type of toilet			
Sanitary latrine with flush	3 (1.7)	3 (1.8)	
Sanitary latrine without flush	163 (93.1)	160 (94.1)	.806
Pucca/pit (without water seal)	8 (4.6)	5 (2.9)	
Kutcha/hanging (fixed place)	1 (0.6)	2 (1.2)	
Religion			
Muslim	173 (98.9)	168 (98.8)	
Hindu	2 (1.1)	2 (1.2)	.977
Employment status			
Unemployed	165 (94.3)	161 (94.7)	.864
Employed	10 (5.7)	9 (5.3)	

### 3.2 Maternal characteristics

Mean maternal age was comparable between groups. A higher proportion of mothers in the control group (58.2%) had not completed secondary education compared with the intervention group (42.9%; *p* = .029), and 26.9% and 17.1% of control and intervention mothers, respectively, had never attended school. Mean body mass index was similar between groups. Approximately three fourths of the mothers in both groups reported that they had previously discussed child feeding during pregnancy. Concerning antenatal care visits, a higher proportion of mothers in the control group (64.7%) had received four or more visits compared with the intervention group (53.7%, *p* < .001). Almost 70% of mothers reported they planned to put their babies to the breast within 1 hr of delivery, and only a few (4.6% of intervention mother and 9.5% of the control mother) reported an intention to give prelacteal feeds to their babies (Table 2).

**Table 2:** Maternal characteristics and antenatal check‐ups during enrolment

Indicator	Intervention (*n* = 175)	Control (*n* = 170)	*p* value
	*n* (%)	*n* (%)	
Mother's age **(**mean ± *SD*)	23.38 ± 4.0	23.54 ± 4.3	.639
Mother's education			
No schooling	29 (17.1)	47 (26.9)	
Primary incomplete	20 (11.8)	28 (16.0)	.029
Secondary incomplete	99 (58.2)	75 (42.9)	
Secondary complete or higher	22 (12.9)	25 (14.3)	
Parity			
One	62 (75.6)	60 (75.0)	.367
Two	17 (20.7)	16 (20.0)	
Three	3 (3.7)	3 (3.7)	
More than three	0 (0.0)	1 (1.3)	
Mother's anthropometry			
Weight (kg)	55.41 ± 9.8	54.63 ± 9.0	.778
Height (cm)	151.19 ± 6.2	150.24 ± 5.4	.951
Body mass index (kg/m^2^)	24.21 ± 3.9	24.19 ± 3.8	.389
Antenatal visits			
None			
One to three	81 (46.3)	60 (35.3)	.038
Four or more	94 (53.7)	110 (64.7)	
Discussion about infant feeding during pregnancy	121 (75.6)	114 (73.5)	.672
Pregnant mother's intention to breastfed	173 (98.9)	166 (97.6)	.585
Intention to initiate breastfeeding			
Within 1 hr	121 (69.1)	111 (65.3)	.446
After 1 hr	54 (30.9)	59 (34.7)	
Intention for prelacteal feeding	8 (4.6)	16 (9.5)	.315

### 3.3 Characteristics of delivery

The characteristics of delivery were similar in both groups. One half of mothers in both groups delivered their babies in health facilities (intervention 52.6% vs. control 51.8%; *p* = .985), whereas approximately one fourth of the mothers in both groups had home deliveries. More than half of the mothers had normal vaginal deliveries, and approximately 31% of both groups had caesarean sections (*p* = .346). Forty‐eight percent of the mothers in the intervention group and 43.7% in the control group were assisted by qualified doctors during delivery (*p* = .695), and over 20% of mothers in both groups were assisted by trained birth attendants (*p* = .681; Table 3).

**Table 3:** Characteristics of deliveries by study group

Indicator	Intervention (*n* = 175)	Control (*n* = 170)	*p* value
	*n* (%)	*n* (%)	
Reported gestational age (mean ± *SD*)	38.76 ± 2.4	38.68 ± 2.3	.379
Birthweight, kg (mean ± *SD*)	2.90 ± 0.5	2.94 ± 0.5	.207
Birth length, cm (mean ± *SD*)	48.58 ± 2.1	48.8 ± 1.7	.177
Low birthweight	21 (12.0)	25 (14.7)	.460
Place of delivery			
Home delivery	47 (26.9)	47 (27.6)	
Facility delivery	92 (52.6)	88 (51.8)	.985
NGO	36 (20.6)	35 (20.6)	
Type of delivery			
Normal	101 (58.7)	9 (53.9)	
Caesarian	54 (31.4)	52 (31.1)	.346
Assisted vaginal delivery	17 (9.9)	25 (15.0)	
Delivery assisted by			
Qualified doctor	83 (48.3)	73 (43.7)	.695
Nurse/midwife/paramedic	16 (9.3)	31 (18.6)	.172
Trained birth attendant	40 (23.3)	37 (22.2)	.681
Untrained birth attendant	33 (19.2)	26 (15.6)	.147
Delivery complications			
Excessive bleeding	1 (0.6)	0 (0.0)	.379
Convulsion	0 (0.0)	1 (0.6)	.596
Prolonged labour	12 (7.0)	6 (3.6)	.381
Ruptured uterus	2 (1.2)	0 (0.0)	.377
High blood pressure	2 (1.2)	5 (3.0)	.429
Infant medical check after delivery			
Yes	91 (55.1)	107 (65.2)	.062
No	74 (44.9)	57 (34.8)	

### 3.4 Effects of the intervention on EIBF and EBF

**Figure 2:**
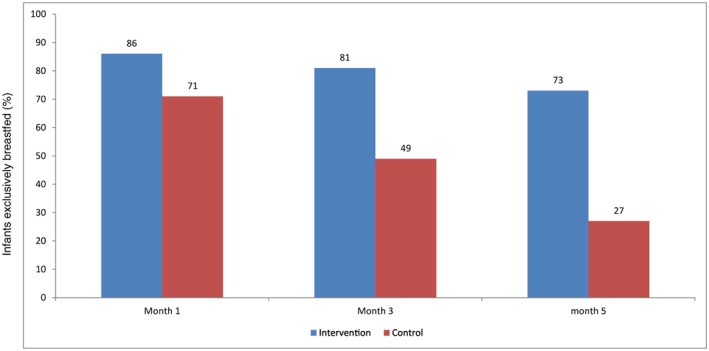
Proportions of infants exclusively breastfed during the first 5 months in the intervention and control areas

The infant feeding practices of the mothers in both the intervention and control groups are shown in Table 4. A significantly higher proportion of mothers in the intervention group reported EIBF compared with the control group (89.1% vs. 77.4%, *p* = .005). More than 95% of mothers in both groups gave colostrum to their babies as their first feed. A higher proportion of neonates in the control group (20/164, 12.2%) received prelacteal feeds compared with the neonates in the intervention group (7/165, 4.2%; *p* = .018). The most common prelacteal feeds given to the babies were water (1/20 in control and 1/7 in intervention area), honey/honey in water (6/20 in control and 2/7 in intervention area), and infant formula (6/20 in control and 3/7 in intervention area). The median duration of EBF for 5 months was 98 days in the intervention group and 56 days in the control group (*p* = .000). The median duration of any breastfeeding in the first year of life was 275 days in the intervention group and 218 days in the control group (*p* = .050). Over 80% of mothers in the intervention group reported peer counsellor was the main person who had advised them to initiate breastfeeding immediately after birth, whereas 48% of the control mothers had received such advice from NGO workers (*p* < .001). Seventy‐six percent of mothers in the intervention group reported the peer counsellor helped them put the child to the breast to initiate breastfeeding, whereas nurses and trained birth attendants helped initiate breastfeeding in the control group (*p* < .001). The proportion of infants exclusively breastfeeding in the intervention group decreased from 86% at 1 month, 81% at 3 months, and 73% at 5 months, whereas the corresponding rates in the control group were 71%, 49%, and 27%. The differences in the EBF practices between the intervention and control group at 5 months were significantly different (*p* < .001; Figure 2).

**Table 4:** Infant feeding patterns among the study participants

Indicator	Intervention (*n* = 165)	Control (*n* = 164)	*p* value^a^
	*n* (%)	*n* (%)	
Initiation breastfeeding			
Initiated BF	147 (89.1)	127 (77.4)	.005
Received colostrum			
Yes	163 (98.8)	159 (96.9)	.248
Prelacteal food given			
Yes	7 (4.2)	20 (12.2)	.018
Type of prelacteal food given	Intervention (*n* = 7)	Control (*n* = 20)	
Honey/sugar water	2/7 (28.6)	6/20 (30.0)	.943
Plain water	1/7 (14.23)	1/20 (5.0)	.419
Commercial infant formula	3/7 (42.9)	6/20 (30.0)	.535
Median duration of EBF (days)	98 (1–150)	56 (1–150)	.000^b^
Median duration of any breastfeeding (days)	275 (1–365)	218 (1–365)	.050^b^
Main person advising about breastfeeding			
Mother of the child	5 (3.0)	16 (9.8)	
Mother	4 (2.4)	12 (7.4)	
Mother in law	0 (0.0)	5 (3.1)	
Other relatives	1 (0.6)	12 (7.4)	<.001
Neighbour	2 (1.2)	1 (0.6)	
Doctor	2 (1.2)	15 (9.2)	
Nurse	8 (4.8)	11 (6.7)	
Trained traditional birth attendant	3 (1.8)	7 (4.3)	
Friends	0 (0.0)	1 (0.6)	
NGO worker	4 (2.4)	78 (47.9)	
Peer counsellor	137 (83.0)	4 (2.4)	
Main person who helped practically with breastfeeding practice			
Mother of the child	6 (3.6)	26 (15.8)	
Mother	10 (6.1)	16 (9.8)	
Husband	0 (0.0)	1 (0.6)	
Mother in law	1 (0.6)	6 (3.7)	
Other relatives	7 (4.2)	14 (8.5)	<.001
Neighbour	0 (0.0)	1 (0.6)	
Doctor	2 (1.2)	2 (1.2)	
Nurse	10 (6.06)	53 (32.3)	
Trained traditional birth attendant	1 (0.6)	27 (16.5)	
Untrained birth attendant	2 (1.2)	10 (6.1)	
NGO worker	0 (0.0)	4 (2.4)	
Peer counsellor	126 (76.4)	4 (2.4)	

*Note*. BF = breastfeeding; EBF = exclusive breastfeeding.

a *p* values calculated using Pearson chi square (*χ* ^*2*^) test or if indicated.

b Mann–Whitney test.

### 3.5 Association of EIBF and EBF with background characteristics

In the adjusted multivariable model, the most significant determinants of a lack of EIBF across the two groups were the mother's education, caesarean section, and low birthweight. The mothers in the control group had a 2.53‐fold higher OR of not initiating breastfeeding immediately compared with the intervention mothers (aOR = 2.53, CI [1.29, 4.97], *p* = .007). Not completing primary education was associated with an increased risk of late initiation of breastfeeding (aOR = 3.74, CI [1.06, 13.14], *p* = .040) compared with mothers with no education. The odds of not receiving early breastfeeding were approximately nine times higher for infants born by caesarean section (aOR: 8.90, CI [4.05, 19.55], *p* < .001) compared with infants who were delivered vaginally. The odds of women practicing EBF for up to 5 months were fivefold higher in the intervention group than the control group (aOR: 5.10, CI [2.89, 9.01], *p* < .001). No other background variables were associated with EBF practices (Tables 5 and 6).

**Table 5:** Factors associated with early initiation of breastfeeding in multivariate analysis

Variable	Crude OR (95% CI)	*p* value	aOR (95% CI)	*p* value
Group				
Intervention	1		1	
Control	2.53 (1.25, 5.14)	.010	2.53 (1.29, 4.97)	.007
Mother's age (years)				
Less than 20	1			
20–29	0.66 (0.38, 1.14)	.119		
30–39	1.61 (0.55, 4.73)	.336		
Mother's education				
No education	1		1	
Primary incomplete	2.86 (0.75, 10.94)	.125	3.74 (1.06, 13.14)	.040
Secondary incomplete	1.74 (0.57, 5.32)	.329	2.02 (0.70, 5.87)	.195
Secondary complete and above	1.34 (0.36, 4.90)	.662	1.66 (0.47, 5.81)	.429
Gestational age				
Term	1		1	
Pre‐term	3.06 (1.39, 6.72)	.005	2.47 (1.18, 5.18)	.017
Type of delivery				
Normal	1		1	
Caesarian	8.48 (2.28, 31.47)	.001	8.90 (4.05, 19.55)	.000
Assisted vaginal delivery	2.37 (0.65, 8.57)	.189	2.85 (1.10, 8.16)	.051
Place of delivery				
Home	1			
Facility based	0.59 (0.12, 2.89)	.004		
NGO based	0.44 (0.08, 2.32)	.335		
Birthweight				
Normal	1			
Low birthweight	2.03 (0.68, 6.26)	.218		
Antenatal check‐up				
One to three	1			
More than three	1.38 (0.68, 2.80)	.369		
Main person who helped with delivery				
Qualified doctor	1			
Nurse/midwife/paramedic	1.26 (0.31, 5.08)	.748		
Trained traditional birth attendant	0.75 (0.14, 3.85)	.727		
Untrained traditional birth attendant	0.37 (0.06, 2.54)	.321		

*Note*. aOR = adjusted odds ratio; OR = odds ratio.

**Table 6:** Factors associated with exclusive of breastfeeding in multivariate analysis

Variable	Crude OR (95% CI)	*p* value	aOR (95% CI)	*p* value
Group				
Control	1		Ref	
Intervention	5.11 (2.89, 9.01)	.000	5.10 (2.89, 9.01)	.000
Mother's age (years)				
Less than 20	1			
20–29	1.02 (0.47, 2.19)	.402		
30–39	0.99 (0.29, 3.41)	.666		
Mother's education				
No education	1			
Primary incomplete	1.42 (0.55, 3.71)	.386		
Secondary incomplete	1.08 (0.58, 2.02)	.801		
Secondary complete and above	1.00 (0.44, 2.27)	.99		
Gestational age				
Term	1			
Pre‐term	1.44 (0.80, 2.60)	.221		
Type of delivery				
Normal	1			
Caesarian	1.47 (0.85, 2.55)	.402		
Assisted vaginal delivery	0.50 (0.22, 1.14)	.666		
Place of delivery				
Home	1			
Facility based	1.50 (0.85, 2.64)	.165		
NGO based	0.58 (0.27, 1.23)	.158		
Birthweight				
Normal	1			
Low birthweight	0.81 (0.40, 1.63)	.559		
Antenatal check‐ups				
One to three	1			
More than three	0.85 (0.51, 1.39)	.510		
Main person who helped with delivery				
Qualified doctor	1			
Nurse/midwife/paramedic	0.30 (0.12, 0.75)	.010		
Trained traditional birth attendant	0.47 (0.25, 0.88)	.018		
Untrained traditional birth attendant	1.00 (0.51, 1.98)	.995		

*Note*. aOR = adjusted odds ratio; OR = odds ratio.

## 4 DISCUSSION

This study provides evidence that peer counselling positively influences EIBF and EBF practices among mothers living in an urban slum. The low prevalence of EBF in the control group and high prevalence of EBF in the intervention group confirm the need for community‐based peer support to promote breastfeeding in urban slum areas. Peer counselling was found to be an effective strategy and led to much higher rates of EBF in the first 5 months compared with the control group.

In our study, we found that the rate of EIBF in the intervention group was 89.1%, which is higher than the values reported for studies in Pakistan and India, but lower than that in Nepal (Khanal, Scott, Lee, Karkee, & Binns, 2015; Patel et al., 2015; Zafar, Fatmi, & Shafi, 2014). There are multiple factors, including maternal education, multiple births, type of delivery, not putting the baby to the breast after birth, and low birthweight, that are associated with a lack of EIBF (Acharya & Khanal, 2015; Gul, Khalil, Yousafzai, & Shoukat, 2014; Khadduri et al., 2008; Sharma & Byrne, 2016). Our study has also shown that maternal education, low birthweight, and caesarean delivery were associated with late initiation of breastfeeding. Due to cultural and traditional norms, family members frequently introduce prelacteals, and health care staffs sometimes advise prelacteals, which conflicts with the advice provided by the peer counsellors. Despite this, the peer counselling significantly reduced prelacteal and postlacteal feeds in the intervention group, in agreement with the results of previous peer counselling studies conducted in Bangladesh and Mexico (Chapman, Damio, Young, & Pérez‐Escamilla, 2004; Haider, Ashworth, Kabir, & Huttly, 2000).

The rate of EIBF in our control group was surprisingly higher than the national average. The last four consecutive Bangladesh Demographic Health Surveys have revealed a significant positive increase in the rate of EIBF over the last decade (26% in 2004 to 43% in 2007). However, the current national rate, 51%, is insufficient to attain the estimated mortality benefits provided by EIBF (Bangladesh Demographic and Health Survey [BDHS], 2004, 2007, 2014). The high prevalence of EIBF in this study may reflect the fact that these disadvantaged mothers only have one option for infant feeding. Similar findings have been reported in Sri Lanka, Tanzania (Adhikari, Khanal, Karkee, & Gavidia, 2014; Exavery, Kanté, Hingora, & Phillips, 2015; Senarath, Dibley, & Agho, 2006), and Nepal.

Approximately one third of mothers in both groups delivered their babies via caesarean section, and these mothers were nine times less likely to initiate breastfeeding immediately after birth. This could be because the newborn baby is not usually put to the mother's breast until the mother has recovered and been transferred from the post‐operative room. An association between caesarean delivery and late initiation of breastfeeding has been demonstrated in several studies (Rowe‐Murray & Fisher, 2002; Senerath et al., 2000). Caesarean delivery has consistently been identified as a major barrier to initiation of breastfeeding immediately after birth (Cakmak & Kuguoglu, 2007; Pandey et al., 2010; Patel et al., 2010). This finding highlights a target where a major intervention could be provided to improve the rate of EIBF (Joshi et al., 2014). This is particularly important, as the percentage of deliveries by caesarean section in Bangladesh has increased over time, from 4% in 2004 to 9% in 2007, 17% in 2011, and 23% in 2014. Mothers who deliver by caesarean section are usually separated from their babies for more than 1 hr after birth, yet this is the best time for successful initiation of EIBF. Even if the babies are not separated from their mothers after surgery, many mothers still decline breastfeeding as they believe the medication they received during the surgical procedure may exert side effects in their babies (Qiu, Zhao, Binns, Lee, & Xie, 2009a); this belief may discourage EIBF (Duong, Binns, & Lee, 2004; Qiu, Zhao, Binns, Lee, & Xie, 2009b).

More than 60% of deliveries still occur at home in Bangladesh (Sarker et al., 2016), so our finding that approximately one third of women delivered their babies at home in both groups implies the likelihood of delivering in a health facility is higher for urban mothers. Our results indicate that mothers who had facility‐based deliveries were less likely to initiate breastfeeding than mothers who delivered at home. This is in contrast to studies in Nepal and Nigeria, which found mothers who deliver in a health facility were more likely to initiate early breastfeeding compared with mothers who delivered at home (Adhikari, Khanal, Karkee, & Gavidia, 2014b; Berde & Yalcin, 2016). The significantly lower proportion of neonates who were given any type of prelacteal feed in the intervention group implies that peer counsellors had a significant influence on reducing prelacteal feeds. Similar findings were reported in earlier studies from India (Kushwaha et al., 2014), Burkina Faso, Uganda, and South Africa (Tylleskar et al., 2011).

Regarding the rate of EBF in developing countries, global data suggest the prevalence of EBF among infants younger than 6 months increased from 33% in 1995 to 39% in 2010 (Haroon et al., 2013). The rate of EBF increased in most in developing regions, with the highest improvements observed in West and Central Africa where the prevalence of EBF more than doubled from 12% in 1995 to 28% in 2010 (Haroon et al., 2013). More modest improvements were observed in South Asia (from 40% in 1995 to 45% in 2010; Cai, Wardlaw, & Brown, 2012). In Bangladesh, the increase in the prevalence of EBF among infants under 6 months was unsatisfactory until the year 2007 (BDHS, 2007). However, the rate of EBF had significantly improved by the last BDHS (2014); the EBF rate is currently 55% (BDHS, 2014). In this study, the prevalence of EBF at 5 months was 27% in the control group and 73% in the intervention group who received peer counselling (*p* < .001).

Two previous RCTs have assessed the ability of community‐based peer counselling to promote EBF, but neither followed up the mothers for as long as 5 months. Haider et al. (2010) were the first to explore the impact of peer counselling in Bangladesh and found that 70% of the mothers in the intervention group practiced EBF but only 6% in the control group practiced EBF at 5 months. A similar approach was used in this trial, and we found 73% of mothers in the intervention group and 27% in the control group practiced EBF at 5 months. The overall nationwide improvements in EBF practices, due to social campaigns, media coverage, programs run by national and international NGOs, and so forth, may possibly explain the higher rate of EBF in the control group in this study compared with that of Haider et al. (2010). The participants in this study lived in urban slum communities, and the EBF rate of the control group was similar to the value of 16% reported in the recent study (Akhtar et al., 2012).

Thus, this study demonstrates one‐to‐one peer counselling may be an effective strategy to improve EIBF and EBF practices in urban slum communities, where a high rate of malnutrition exists and inappropriate breastfeeding practices are prominent (Shakya et al., 2017). Our peer counsellors delivered a regular service on a part‐time (half‐day) basis and received a monthly honorarium of approximately US $43. The counsellors also made additional visits if necessary and conducted fortnightly group sessions with the mothers and other family members. In a lower resource setting, fewer visits combined with group counselling may be effective to increase the rates of EIBF and EBF (Haider et al., 2000). Governments and NGOs could even consider training community health volunteers to provide group or peer support on infant and young child feeding practices to the mothers and caregivers.

One strength of this study is its randomized design, which minimized selection bias (Sibbald & Roland, 1998). Moreover, the power of randomization provides excellent internal validity, which is one of the greatest strengths of RCTs. Randomization ensures that the level of exposure to the treatment of interest is the only differentiating factor between the two arms. Strength of this study is that the intervention combined individual counselling with fortnightly group sessions. Including pregnant and lactating mothers and family members in counselling and practical demonstrations has also been proven to be effective (Sibbald & Roland, 1998).

One limitation of this randomized study is that it was not double blinded, and the interviewer knew which clusters were included in the intervention group. To prevent interviewer bias, the questions related to peer counsellor contact were asked to the mothers at the end of each follow‐up interview session. Additionally, the generalizability (external validity) of this study may be limited (Meyer, 2010), as there was a high dropout rate during the trial (25% in the intervention group and 16% in the control group). In most urban slums in Bangladesh, including Mirpur, people often move from one slum to another in search of better employment, cheaper accommodation, or in response to political unrest. Moreover, many working mothers who formerly resided in urban slums often quit their jobs to take care of their children and migrate back to rural areas. This phenomenon could explain the high dropout rate and loss to follow‐up in this study.

In summary, we found that peer counselling was an effective method of promoting EIBF as well as EBF among urban women living in a slum in Dhaka, Bangladesh. This result is very promising, as this slum community has exceedingly low rates of EBF and a strong preference for formula, even if mothers choose to breastfeed their infants (Chapman et al., 2004; Perez‐Escamilla et al., 1998). We have shown the potential of peer counselling to promote EIBF and EBF among women residing in urban slum areas.

## 5 CONCLUSION

Peer counselling positively influenced the initiation of breastfeeding within 1 hr of birth and the duration of EBF and reduced prelacteal feeding among urban women living in a slum in Dhaka, Bangladesh. This study highlights the need to target the promotion of optimal breastfeeding practices to expectant mothers and mothers who deliver through caesarean section. Furthermore, peer counselling support to support EBF should be continued for at least first months of delivery.

## AVAILABILITY OF DATA AND MATERIALS

The dataset has been uploaded as Additional File 1 (EBIF.dta).

## CONFLICTS OF INTEREST

The authors declare that they have no conflicts of interest.

## CONTRIBUTIONS

GA was responsible for the overall design and implementation of the study, for securing funding, for training and project management, and for data management and analysis. MJD and IK helped design the study and provided technical assistance throughout all stages of the project. BN helped train the interviewers. MK, NP, and Md. AH helped with daily project management, data management, and analysis. All authors are responsible for the final content of this manuscript and have read and approved the final version.
